# *Prevotella intermedia* boosts OSCC progression through ISG15 upregulation: a new target for intervention

**DOI:** 10.1007/s00432-024-05730-5

**Published:** 2024-04-21

**Authors:** Yao Qin, Zhiyuan Li, Ting Liu, Jingjing Ma, Hong Liu, Yifan Zhou, Shuai Wang, Lei Zhang, Qiao Peng, Pei Ye, Ning Duan, Wenmei Wang, Xiang Wang

**Affiliations:** Nanjing Stomatological Hospital, Affiliated Hospital of Medical School, Institute of Stomatology, Nanjing University, 30 Zhongyang Road, Nanjing, 210008 China

**Keywords:** *Prevotella intermedia*, Oral squamous cell carcinoma, Interferon-stimulated gene 15, Tumor microenvironment, RNA sequencing, Transcriptomics

## Abstract

**Purpose:**

Periodontitis-associated bacteria, such as *Porphyromonas gingivalis* and *Fusobacterium nucleatum*, are closely linked to the risk of oral squamous cell carcinoma (OSCC). Emerging studies have indicated that another common periodontal pathogen, *Prevotella intermedia* (*P. intermedia*), is enriched in OSCC and could affect the occurrence and progression of OSCC. Our aim is to determine the effects of *P. intermedia* on the progression of OSCC and the role of antibiotics in reversing these effects.

**Methods:**

In this study, a murine xenograft model of OSCC was established, and the mice were injected intratumorally with PBS (control group), *P. intermedia* (*P.i* group), or *P. intermedia* combined with an antibiotic cocktail administration (*P.i* + ABX group), respectively. The effects of *P. intermedia* and ABX administration on xenograft tumor growth, invasion, angiogenesis, and metastasis were investigated by tumor volume measurement and histopathological examination. Enzyme-linked immunosorbent assay (ELISA) was used to investigate the changes in serum cytokine levels. Immunohistochemistry (IHC) was adopted to analyze the alterations in the levels of inflammatory cytokines and infiltrated immune cells in OSCC tissues of xenograft tumors. Transcriptome sequencing and analysis were conducted to determine differential expression genes among various groups.

**Results:**

Compared with the control treatment, *P. intermedia* treatment significantly promoted tumor growth, invasion, angiogenesis, and metastasis, markedly affected the levels of inflammatory cytokines, and markedly altered M2 macrophages and regulatory T cells (Tregs) infiltration in the tumor microenvironment. However, ABX administration clearly abolished these effects of *P. intermedia*. Transcriptome and immunohistochemical analyses revealed that *P. intermedia* infection increased the expression of interferon-stimulated gene 15 (ISG15). Correlation analysis indicated that the expression level of ISG15 was positively correlated with the Ki67 expression level, microvessel density, serum concentrations and tissue expression levels of inflammatory cytokines, and quantities of infiltrated M2 macrophages and Tregs. However, it is negatively correlated with the quantities of infiltrated CD4^+^ and CD8^+^ T cells.

**Conclusion:**

In conclusion, intratumoral *P. intermedia* infection aggravated OSCC progression, which may be achieved through upregulation of ISG15. This study sheds new light on the possible pathogenic mechanism of intratumoral *P. intermedia* in OSCC progression, which could be a prospective target for OSCC prevention and treatment.

**Supplementary Information:**

The online version contains supplementary material available at 10.1007/s00432-024-05730-5.

## Introduction

Head and neck squamous cell carcinoma (HNSCC) is the sixth most common cancer worldwide, with high rates of metastasis, recurrence and mortality. Oral squamous cell carcinoma (OSCC) accounts for over 90% of all HNSCCs, and OSCC patients have a 5-year survival rate of < 50%, which imposes an enormous global health burden (Chinn and Myers 2015). More than 350,000 new cases of OSCC and 175,000 OSCC-related deaths occur each year (Bray et al. 2018). Possible risk factors include viral infection, fungal infection, and chronic periodontitis (Mallika et al. 2020). However, at present, the specific pathogenesis of OSCC is still not clear.

It is estimated that 20% of all fatal cancers in humans are caused by microorganisms. The promoting or suppressing effects of oral microbes on OSCC have been studied. In particular, some periodontitis-related bacteria have been suggested to be closely associated with the development of OSCC (Polak et al. 2009; Wen et al. 2020). *P. intermedia*, a Gram-negative anaerobic bacterium, is recognized as a common periodontal pathogen. It is associated with esophageal squamous cell carcinoma (ESCC) and colorectal cancer (CRC) (Kawasaki et al. 2021; Lo et al. 2022). Our previous study showed that the *P. intermedia* abundance gradually increases across stages of oral carcinogenesis and may play an important role in the progression of OSCC (Heng et al. 2022). However, to date, few studies have investigated the specific role of *P. intermedia* in OSCC development, and the related mechanisms are still unclear.

**Fig. 1:**
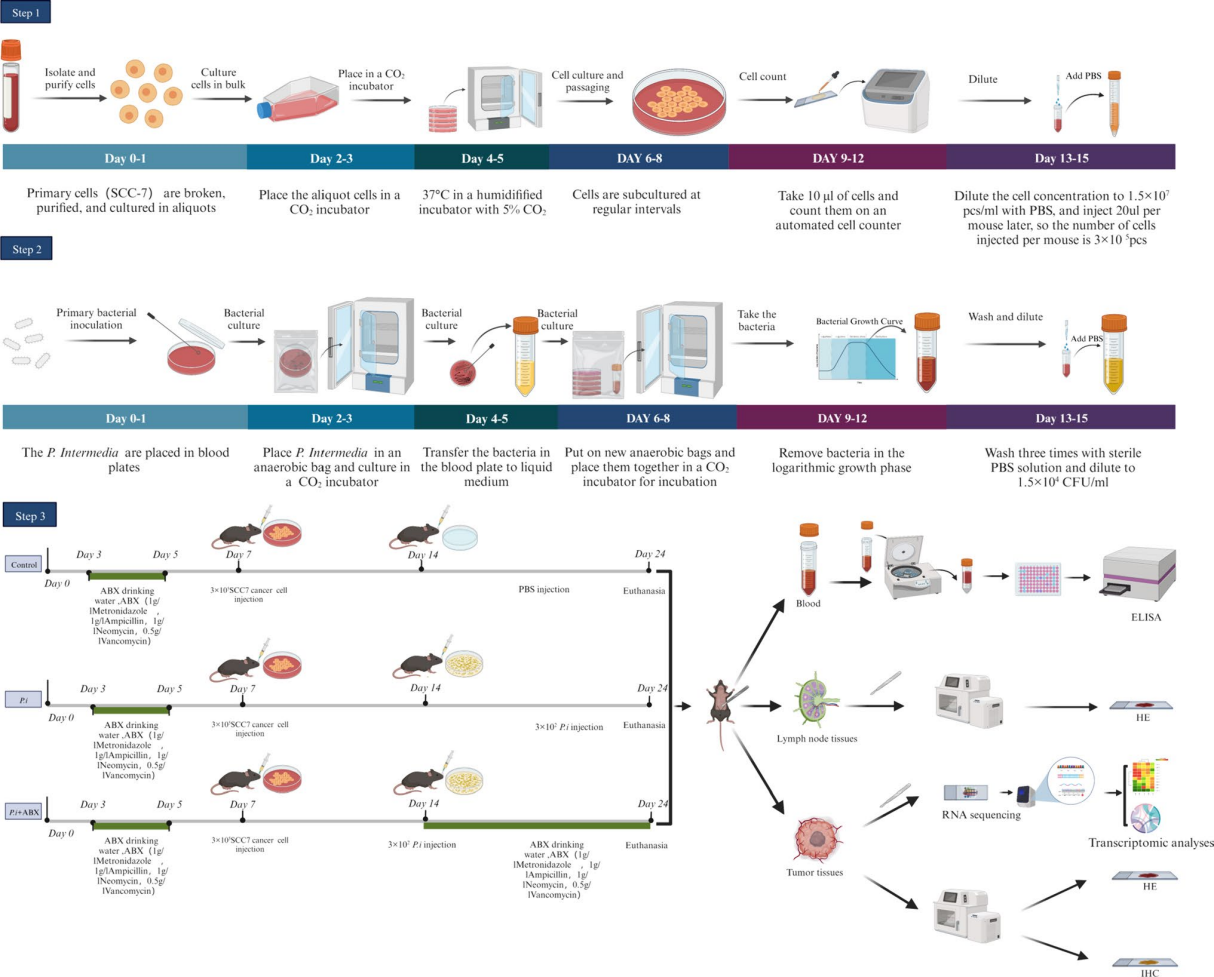
Flowchart of the experimental design. Preparation of SCC-7 cells, preparation of *P. intermedia* and mouse xenograft model of OSCC

This study aimed to determine the effects of *P. intermedia* on the progression of OSCC and the role of antibiotics in reversing these effects. To this end, a murine SCC-7 tumor-bearing model with or without *P. intermedia* intratumoral injection was used in our study. Tumor growth, invasion, angiogenesis, and neck node metastasis were analyzed through the macroscopic observation and hematoxylin–eosin (H&E) staining. The serum and tissue levels of inflammatory cytokines and immune cell infiltration were determined using enzyme-linked immunosorbent assay (ELISA), immunohistochemistry (IHC), and immunofluorescence (IF). Differential expression genes analysis was performed according to RNA sequencing and transcriptome analysis (Fig. 1).

## Materials and methods

### Cells and culture conditions

The murine OSCC cell line SCC-7 was obtained from Cellcook Biotech Co., Ltd. (Guangzhou, China). Cells were grown in Dulbecco’s modified Eagle’s medium (DMEM; HyClone, Logan, UT, USA) supplemented with 10% fetal bovine serum (FBS; Gibco, Grand Island, NY, USA) at 37 °C in a humidified incubator with 5% CO_2_. SCC-7 cells were used at 75% confluence. In this study, 3 × 10^5^ SCC-7 cells were used to establish xenograft tumors in mice.

### Bacteria and culture conditions

*P. intermedia* (ATCC 25611) was cultured on brain heart infusion (BHI; BD, Basingstoke, USA) blood agar plates supplemented with hemin (5 mg ml^−1^), menadione (1 mg ml^−1^) and 5% defibrinated sheep blood under anaerobic conditions at 37 °C overnight. Bacteria were harvested in the late exponential growth phase by centrifugation for 10 min at 4000× g and 4 °C and were washed three times with sterile phosphate-buffered saline (PBS) before use. Then, we used PBS to dilute the bacterial concentration to 1.5 × 10^4^ colony-forming units (CFU)/ml. A mixture containing 3 × 10^2^ CFU of *P. intermedia* was used in animal experiments.

### Animal model and treatments

Female C57BL/6 mice (6 weeks old) were obtained from Sino-British SIPPR/BK Lab Animal Ltd. (Shanghai, China). All of the murine experiments were performed in accordance with the ethical guidelines for animal experimentation and were approved by the Ethics Committee of Nanjing Stomatological Hospital, Affiliated Hospital of Medical School, Nanjing University (IRB approval number: 2018NL-008 (KS) & NJSH-2023NL-18) and the Animal Ethical and Welfare Committee of Nanjing University (IACUC-D2202108 & IACUC D2303084). All mice were acclimatized to the experimental facility for three days to relieve stress and were fed an antibiotic cocktail (ABX) (1 g/L ampicillin, 1 g/L metronidazole, 1 g/L neomycin, and 0.5 g/L vancomycin) (Solarbio, China), which was dissolved in the drinking water, for 3 days before tumor cell inoculation.

The mice were randomly divided into three groups: control group, *P.i* group, and *P.i* + ABX group. Then, the mice were anesthetized by intraperitoneal injection of 1% pentobarbital sodium. A total of 3 × 10^5^ SCC-7 cells suspended in 20 μL of PBS were injected into the submucosa of the right bucca of each mouse (Li et al. 2023). One week after cell inoculation, tumors were visible in the bucca of all mice. Then, for the *P.i* group and *P.i* + ABX group, *P. intermedia* were suspended (3 × 10^2^ CFU in 20 μL of PBS medium) and injected into the tumor of each mouse. For the *P.i* + ABX group, the mice were also fed an antibiotic cocktail (ABX) (1 g/L ampicillin, 1 g/L metronidazole, 1 g/L neomycin, and 0.5 g/L vancomycin) (Solarbio, China) dissolved in the drinking water. For the control group, the mice were injected with 20 μL of PBS medium only. After ten days of *P. intermedia* infection, the mice in all groups were scheduled for sacrifice, and their tumors and lymph nodes were harvested. Tumor volumes and weights were evaluated. Tumor volumes were determined using the following equation: tumor volume (mm^3^) = (minimum diameter)^2^ × (maximum diameter)/2. The experimental process for this study is shown in Fig. 1.

### Histochemistry, immunohistochemistry, and immunofluorescence

H&E staining was performed using formalin-fixed, paraffin-embedded tumor and cervical lymph nodes following the instructions in the product manual. IHC and IF staining were also conducted according to the instructions. Sections were observed under an inverted microscope. Protein expression indicated by IHC and IF staining was assessed in 3 different randomly selected regions of interest (ROIs). IHC staining of Interleukin (IL)-6, IL-17, Vascular endothelial growth factor A (VEGF-A), and ISG15 was evaluated by calculating the mean IHC scores using ImageJ software (version 1.8.0_172, USA). Based on the intensity, staining was divided into four grades: 0 (no staining), 1 (light yellow staining), 2 (light brown staining), and 3 (tan staining). Additionally, based on the proportion of positive cells, staining was divided into five grades: 0 (0–4% positive cells), 1 (5–25%), 2 (26–50%), 3 (51–75%), and 4 (76–100%). The overall immunostaining score was calculated as follows: immunoreactivity score (IRS) = percentage score × intensity score (Guo et al. 2021). IHC or IF staining of Ki67, Forkhead box protein 3 (FOXP3), CD4, CD8, F480, and CD206 was scored by determining the proportion of highly positive cells (Wang et al. 2023b). All slides were independently scored by pathologists who were blinded to the experimental data. A high level of concordance (90%) was achieved.

### Enzyme-linked immunosorbent assay

ELISA was performed on serum from euthanized mice to measure the concentrations of IL-17, IL-6, tumor necrosis factor (TNF)-α, and interferon (IFN)-γ. All ELISA kits were purchased from Multiscience Biotechnology Co., Ltd. (Hangzhou, Zhejiang, China).

### RNA sequencing and transcriptome analysis

Total RNA was extracted using TRIzol reagent (Invitrogen, CA, USA) according to the manufacturer’s protocol. Then, libraries were constructed using the VAHTS Universal V6 RNA-seq Library Prep Kit according to the manufacturer’s instructions. Transcriptome sequencing and analysis were conducted by OE Biotech Co., Ltd. (Shanghai, China). The FPKM value of each gene was calculated, and the read count of each gene was obtained by HTSeq-count (Anders Pyl and Huber 2015).

Differential expression analysis was performed using DESeq2 (Love Huber and Anders 2014). *Q* value < 0.05 and fold change > 1 were set as the threshold criteria for identifying significantly differentially expressed genes (DEGs). Hierarchical cluster analysis of the DEGs was performed using R (v 3.2.0) to demonstrate the expression patterns of genes in different groups and samples.

Based on the hypergeometric distribution, GO term (2019), KEGG pathway (Kanehisa et al. 2008), Reactome, and WikiPathways enrichment analyses were performed with the DEGs to screen for significantly enriched terms and pathways using R (v 3.2.0). Gene set enrichment analysis (GSEA) was performed using GSEA software (Mootha et al. 2003; Subramanian et al. 2005). The analysis used a predefined gene set and the genes were ranked according to the degree of differential expression in the two types of samples. Then, we tested whether the predefined gene set was enriched at the top or bottom of the ranking list.

### Protein–protein interaction network construction and identification of hub genes

To further explore the interactions among the common differentially expressed genes (co-DEGs), a protein–protein interaction network (PPI) of the co-DEGs was constructed using the Search Tool for the Retrieval of Interacting Genes (STRING) (http://string-db.org/) database (Szklarczyk et al. 2019). The PPI network was visualized using Cytoscape software (Kohl Wiese and Warscheid 2011). CytoHubba was used to identify the hub genes in the PPI network of the co-DEGs, and the shade of color corresponds to the criticality of the hub gene.

### Statistical analysis

The R ggpubr package was used to perform statistical analyses. Data were expressed as the means ± standard deviations (SDs) from at least 3 independent experiments. The statistical significance of differences among three groups was determined by ordinary one-way analysis of variance (ANOVA). The level of statistical significance was set at *P* ≤ 0.05. GraphPad Prism 6.0 (Graph Software Inc.) was applied for graphing.

## Results

### *P. intermedia* promoted tumor growth and invasion, while the administration of ABX abolished these effects

**Fig. 2:**
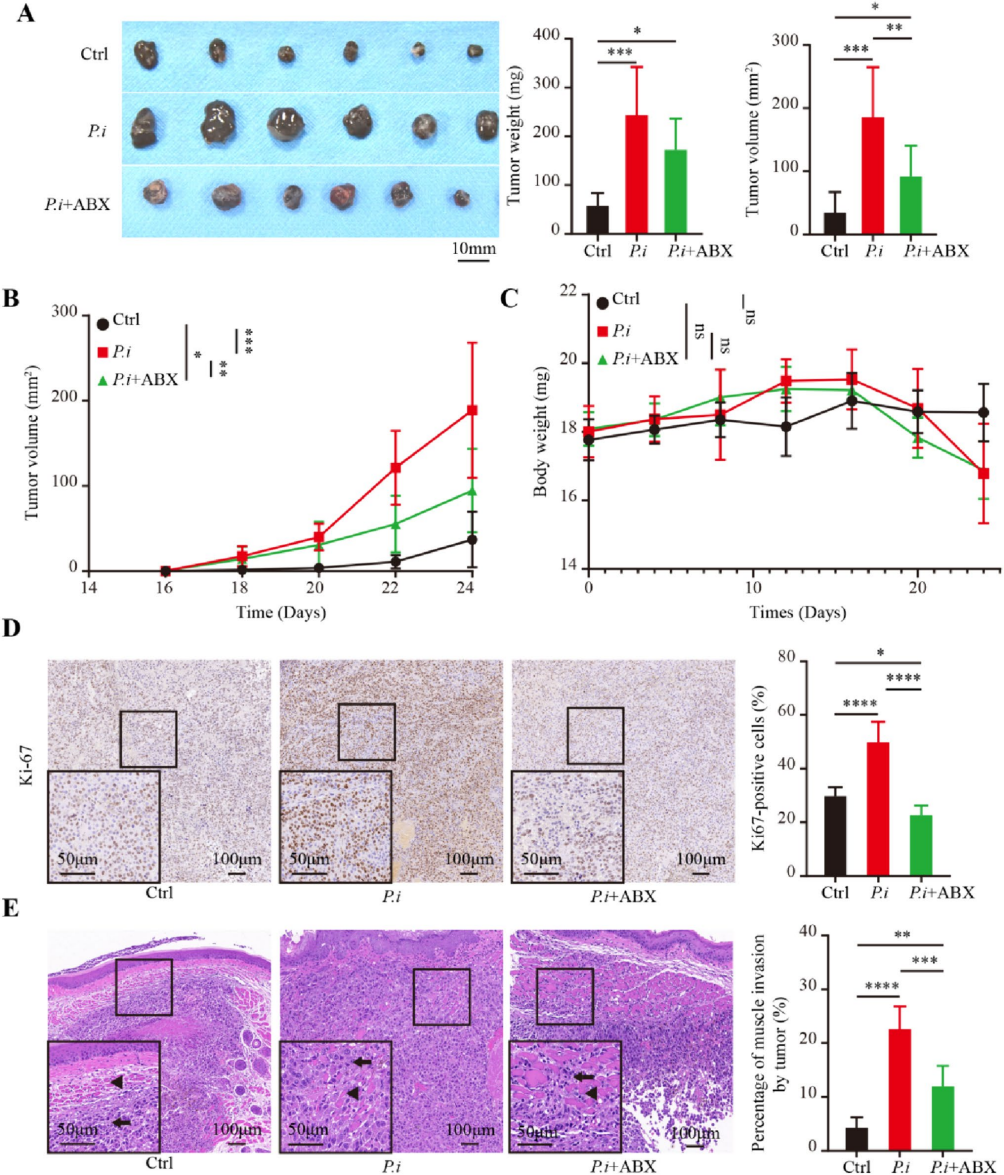
Effects of *P. intermedia* and ABX treatments on the tumor volume, weight, and histopathological features in the mouse xenograft model of OSCC. **A** Macroscopic observation of tumors. Quantitation of the weight and volume of the three groups is on the right. **B** Tumor volume changes. **C** Body weight changes. **D** Representative IHC images for Ki67. Quantitation is on the right. **E** Microscopic observation of tumors in the three groups. The black arrows indicate the tumor and triangles indicate the muscle. Quantitation is on the right. The data are shown as the means ± SDs and were analyzed using one-way ANOVA. *P < 0.05; **P < 0.01; ***P < 0.001; ****P < 0.0001

We established a xenograft model in mice and explored the effects of *P. intermedia* on OSCC development in vivo. SCC-7 cells were implanted in the right buccal submucosa of mice. After tumor formation, *P. intermedia* was injected into the tumors. Macroscopic observation and morphometric analysis revealed that infection with *P. intermedia* markedly promoted the growth of OSCC tumors. Tumor weight and tumor volume in the *P.i* group were significantly higher than those in the control group (Fig. 2A–B). In the *P.i* + ABX group, we found that tumor growth was markedly retarded after ABX administration (Fig. 2A–B). However, the changes in mouse body weight during the experiment were small (Fig. 2C).

IHC analysis revealed that Ki67 expression was markedly augmented in the *P.i* group, indicating that *P. intermedia* infection can enhance the proliferative activity of SCC-7 cells in vivo. However, after the administration of ABX, the Ki67 expression level was significantly decreased (Fig. 2D). Moreover, in the *P.i* group, there was disseminated invasion of tumor cells into the muscle and discontinuous muscle alignment (Fig. 2E). In the *P.i* + ABX group, there were fewer tumor cells invading the muscle layer than in the *P.i* group (Fig. 2E). These results suggested that *P. intermedia* promoted OSCC growth and invasion in vivo, whereas ABX administration inhibited tumor growth and invasion to some extent.

### *P. intermedia* facilitated tumor angiogenesis, while the administration of ABX abolished this effect

**Fig. 3:**
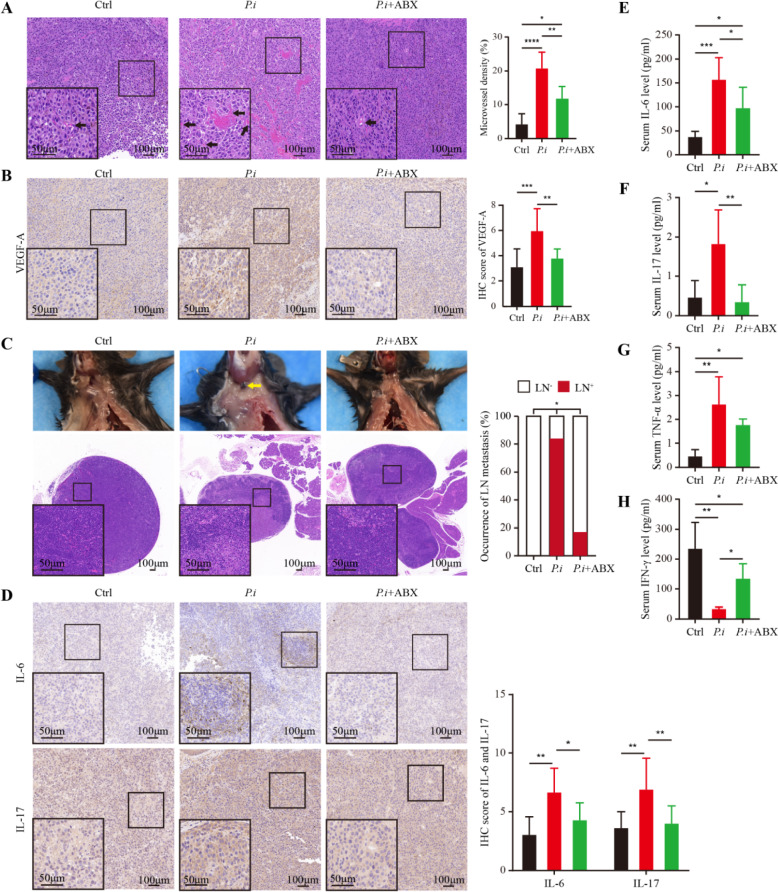
Effects of *P. intermedia* and ABX treatments on tumor angiogenesis, lymph node metastasis, and inflammatory cytokine levels in a mouse xenograft model of OSCC. **A** The angiogenesis in xenografts and the microvessel density comparison among the three groups. Quantitation is on the right. **B** Representative IHC images for VEGF-A. Quantitation is on the right. **C** Representative macroscopic and microscopic images of neck lymph node metastasis. Quantitation is on the right. **D** Representative IHC images for IL-17 and IL-6. Quantitation is on the right. **E**–**H** Measurement of serum concentrations of IL-6, IL-17, TNF-α and IFN-γ in mice. The data are shown as the means ± SDs and were analyzed using one-way ANOVA. LN: Lymph node; +: positive; −: negative. *P < 0.05; **P < 0.01; ***P < 0.001; ****P < 0.0001

To evaluate the effect of *P. intermedia* infection on tumor vascularization, we analyzed the tumor vasculature in the three groups. The H&E staining results showed an abundance of dilated blood vessels in the *P.i* group. Following the administration of ABX, the number of dilated blood vessels decreased, and the size of the vessels decreased. Our analysis results indicated that the microvessel density in the *P.i* group was significantly higher than that in the control group, while the microvessel density in the *P.i* + ABX group was markedly lower than that in the *P.i* group (Fig. 3A). Furthermore, we identified vascularization by evaluating the expression of VEGF-A in the tumor microenvironment (TME). Our results indicated that the level of VEGF-A expression in the *P.i* group was significantly higher than that in the control group, while the expression level of VEGF-A in the *P.i* + ABX group was markedly lower than that in the *P.i* group (Fig. 3B). These data indicated that *P. intermedia* infection promoted vascularization and vessel sprouting. Thus, *P. intermedia* infection might play a crucial role in promoting tumor vascularization in OSCC and that the administration of ABX may have a function in reversing this influence on tumor vascularization.

### *P. intermedia* accelerated lymph node metastasis, while the administration of ABX abolished this effect

The H&E staining results of neck lymph nodes showed that five in six of the mice in the *P.i* group possessed lymph node metastases, while the rate of lymph node metastasis in the *P.i* + ABX group was only one in six. In the control group, none of the mice possessed lymph node metastases. In the gross images, darkened regions in the lymph nodes were observed in the mice with neck lymph node metastases. Through H&E staining, metastatic tumor cells, sharply distinguished from lymphocytes, were observed in the neck lymph nodes (Fig. 3C). Then, we compared the occurrence of neck lymph node metastasis among the three groups. The analysis results indicated that there were clear differences in neck lymph node metastasis among the groups (Fig. 3C).

### *P. intermedia* altered the serum concentrations and tissue expression levels of inflammatory cytokines, while the administration of ABX abolished these effects

Our study also showed that *P. intermedia* infection contributed to inflammatory responses in the SCC-7 tumor-bearing model. Regarding the expression levels of proinflammatory cytokines in tumor tissues, the relative IHC scores of IL-17 and IL-6 were clearly increased after *P. intermedia* infection compared with those in the control group (Fig. 3D). The relative IHC scores of IL-17 and IL-6 were markedly decreased after the administration of ABX.

Moreover, to evaluate the effects of *P. intermedia* infection on the serum concentrations of inflammatory cytokines in OSCC, we collected mouse serum and performed ELISA. The ELISA results showed that *P. intermedia* infection also significantly elevated the serum concentrations of IL-6, IL-17, and TNF-α. Interestingly, we found that *P. intermedia* infection markedly reduced the serum concentration of IFN-γ, which is a pleiotropic cytokine involved in antiviral responses, immune surveillance, inhibition of cell proliferation, and tumor suppression (Wei et al. 2011) (Fig. 3E–H).

### *P. intermedia* modified immune cell infiltration, while the administration of ABX abolished this effect

**Fig. 4:**
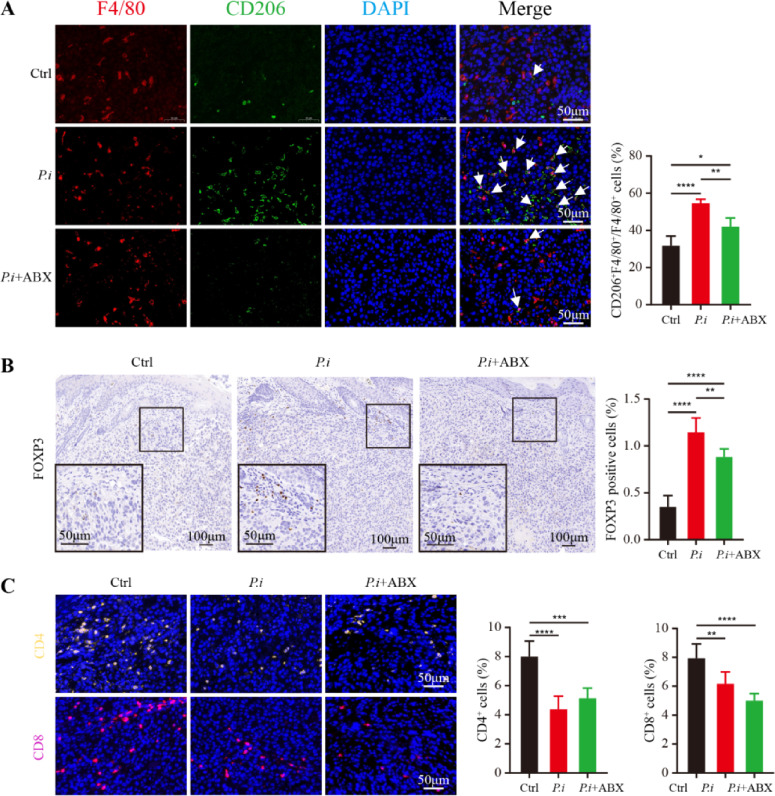
Effects of *P. intermedia* and ABX treatments on immune cell infiltration in the mouse xenograft model of OSCC. **A** Representative IF images for F4/80 and CD206 and the comparison of M2 macrophage (F4/80^+^CD206^+^) percentages among the three groups. **B** Representative IHC images for FOXP3. **C** Representative IF images for CD4^+^ and CD8^+^ T cells. Quantitation is on the right. The data are shown as the means ± SDs and were analyzed using one-way ANOVA. *P < 0.05; **P < 0.01; ***P < 0.001; ****P < 0.0001

To investigate the composition of tumor-infiltrating immune cells, IHC and IF staining were used to determine the proportions of M2 macrophages, regulatory T cells (Tregs), and CD4^+^ and CD8^+^ T cells. Macrophages are polarized into two phenotypes: M1 and M2. In the TME, tumor-associated macrophages (TAMs) often exhibit M2 polarization and are associated with the malignant transformation of tumor cells and poor prognosis of patients (Chen et al. 2020). The FOXP3 is the main marker of Tregs and dominantly determines the function of Tregs (Wang et al. 2023a). We conducted immunolocalization analysis based on F4/80 and CD206 and found that there were significantly more M2 macrophages (F4/80^+^CD206^+^) in the *P.i* group than in the control group (Fig. 4A). However, the quantity of infiltrated M2 macrophages in the *P.i* + ABX group was markedly lower than that in the *P.i* group (Fig. 4A). Similarly, the IHC results also showed that the quantity of infiltrated Tregs was markedly increased in the *P.i* group but clearly decreased after the administration of ABX (Fig. 4B). Additionally, the quantities of infiltrated CD4^+^ and CD8^+^ T cells were significantly reduced in the *P.i* group and *P.i* + ABX group compared to the control group (Fig. 4C). Taken together, these data indicated that *P. intermedia* could affect the infiltration of immune cells in OSCC tissues, which contributes to an immunosuppressive TME.

### *P. intermedia* increased ISG15 expression, while the administration of ABX abolished this effect

**Fig. 5:**
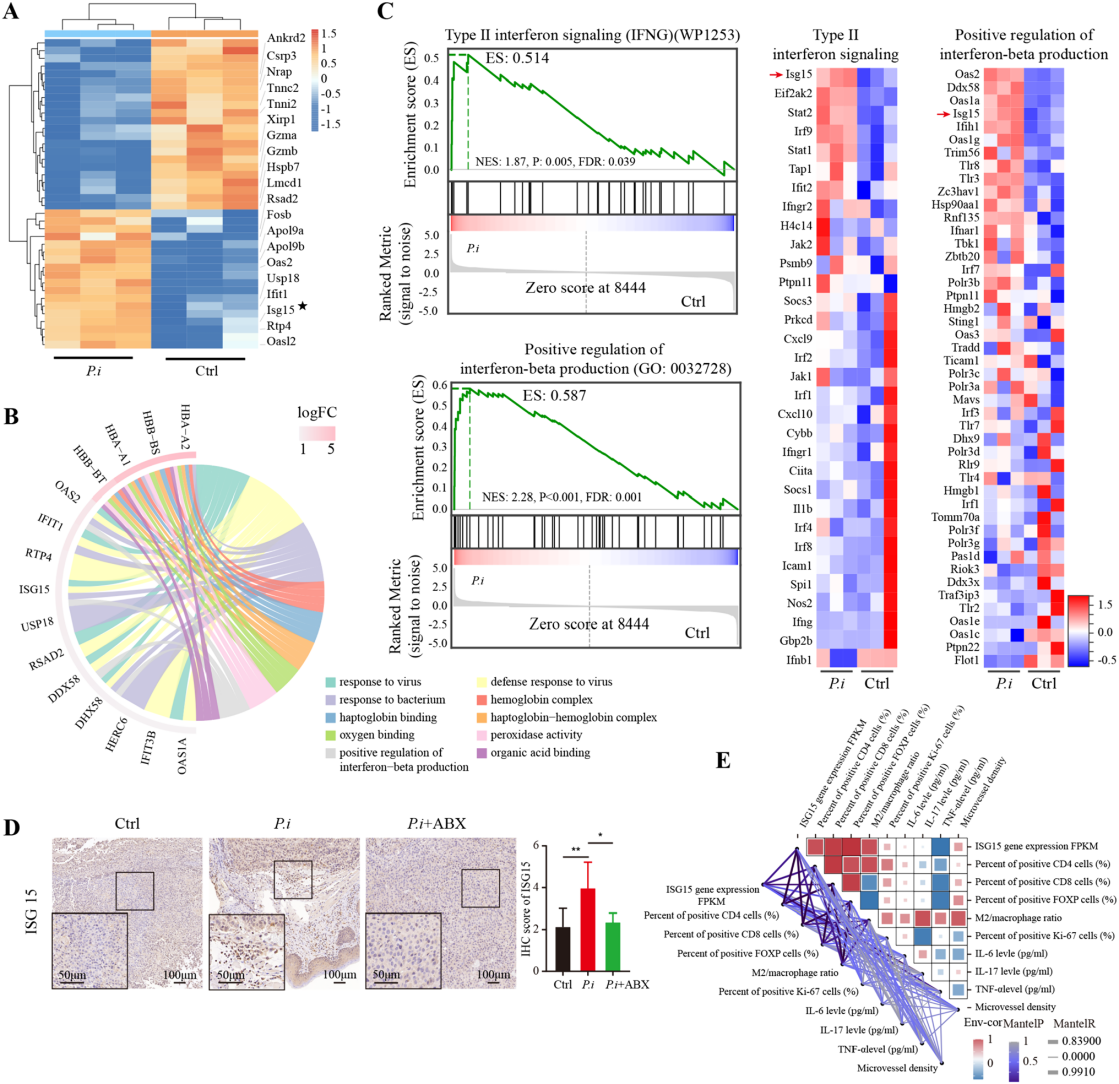
Transcriptome profiling with the RNA sequencing results. **A** Clustered heatmaps of differentially expressed genes between the control group and *P.i* group. Horizontal coordinates represent samples and vertical coordinates represent different genes. Orange represents up-regulated differentially expressed genes and blue represents down-regulated differentially expressed genes. **B** Correlations between functions are shown on chord diagrams. The arcs on the outer circle indicate the categories of significantly different functions. The colored lines indicate correlations within the various functions. **C** GSEA-based Gene Ontology (GO) analysis of the representative gene set and GSEA software-generated heatmaps showing the highly enriched genes. NES, normalized enrichment score. FDR, false discovery rate. **D** Representative IHC images for ISG15. Quantitation is on the right. **E** Correlation plots showing the Pearson correlation coefficients for all tumor-related factors analyzed in the present study

We next investigated the molecular mechanisms of *P. intermedia* in depth by mRNA sequencing. In total, 18 DEGs were significantly up-regulated and 22 DEGs were significantly down-regulated in the *P. intermedia*-treated tumor tissues compared with the control tumor tissues (Supplementary Fig. 1 and Table 1). A clustered heatmap was generated to visually present the changes in the expression of the up-regulated and down-regulated DEGs and revealed the distribution of the gene expression data of each subset (Fig. 5A). Further analysis of the DEGs showed that the majority of the top 10 up-regulated genes belonged to the family of interferon-stimulated genes (ISGs). Among these detected ISGs, we focused on ISG15 as a gene of potential interest, as it encodes a protein that can function intracellularly to modify the expression of cytoplasmic and nuclear proteins. ISG15 can also be secreted from activated cells as free ISG15 and has been shown to play putative roles in other solid tumors, such as pancreatic ductal adenocarcinoma, hepatocellular carcinoma, and nasopharyngeal carcinoma (Burks et al. 2019; Chen et al. 2020; Li et al. 2014).

We also observed an increase in the level of the ISG15 deconjugating enzyme USP18 (Fig. 5A), which functions to deconjugate ISG15 from its target proteins, thus increasing the overall amount of monomeric ISG15 (Liu et al. 1999). USP18 is also associated with tumorigenesis, and studies have shown that USP18 expression is up-regulated in various tumors (Mustachio et al. 2017). The administration of ABX partly reversed the *P. intermedia*-induced changes in gene expression in tumors (Supplementary Fig. 2 and Table 2). The chord diagram shows the pathways and functions closely related to the ISG15 gene, which were defense response to virus, response to bacterium and positive regulation of interferon-beta production (Fig. 5B).

To identify the possible ISG15-associated pathways in OSCC, GSEA was conducted. The heatmap and GSEA enrichment plot showed that ISG15 expression may be associated with the pathways Type II interferon signaling, Positive regulation of Interferon-beta production, ISG15 antiviral mechanism, RIG-I-like receptor signaling pathway, Antiviral mechanism by IFN-stimulated genes, and Negative regulation of viral genome replication (Fig. 5C and Supplementary Fig. 3). We confirmed the transcriptomic results by IHC analysis of ISG15. The IHC results showed a significant increase in ISG15 protein expression in the *P.i* group (Fig. 5D). Pearson correlation analyses were conducted to evaluate correlations between ISG15 expression and tumor-associated factors (Fig. 5E). Correlation analysis showed that ISG15 gene expression was positively correlated with the quantities of infiltrated M2 macrophages and Tregs. ISG15 gene expression was also positively correlated with the concentrations of IL-6, IL-17, and TNF-α in mouse serum and the microvessel density in tumors.

### Protein‒protein interaction network construction and identification of hub genes

**Fig. 6:**
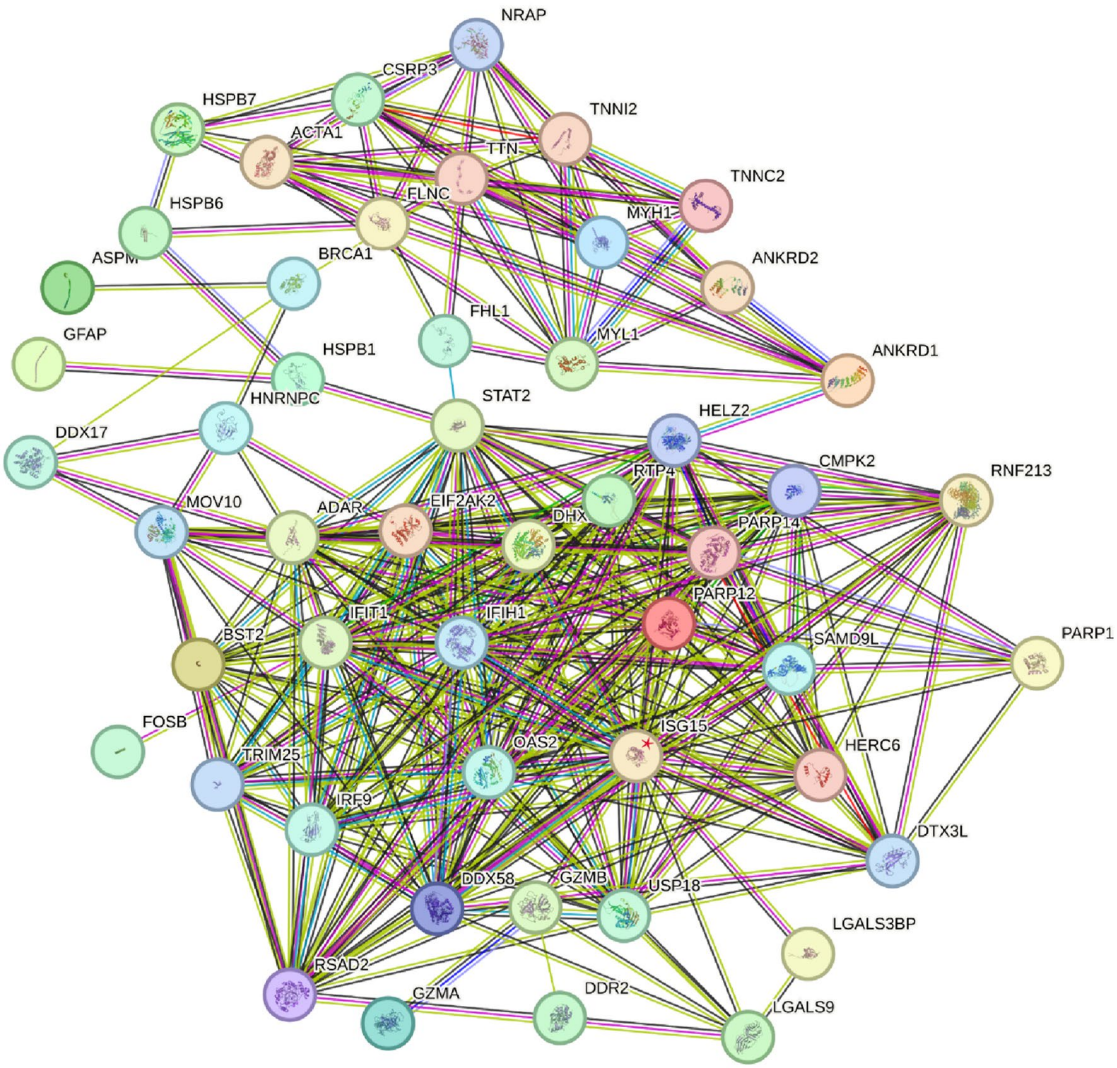
A PPI network is built to explore the interaction among co-DEGs between the control and *P.i* group. Using the Network Analyzer plug-in to modify the PPI network. The network nodes represent proteins and the edges represent both functional and physical protein associations. Different line colors indicates the type of interaction evidence. 53 nodes and 327 edges were displayed

In total, 84 co-DEGs obtained from the aforementioned analyses were input into the STRING platform to screen for their interacting proteins, and the results were imported into Cytoscape software to construct a PPI network (Fig. 6). The network nodes represent proteins and the edges represent both functional and physical protein associations. Different line colors indicates the type of interaction evidence. In addition, the top 10 genes were identified as core genes by calculating the tightness of the connection of the node using the cytoHubba plug-in (Supplementary Fig. 4). STAT2, EIF2AK2, DHX58, USP18, CMPK2, ISG15, IFIH1, OAS2, RSAD2, and DDX58 were identified as the hub genes of the network. ISG15, CMPK2, IFIH1, DDX58 and USP18 were associated with the most significant module. There were strong interactions among these hub genes that may affect the pathophysiological process of OSCC.

## Discussion

**Fig. 7:**
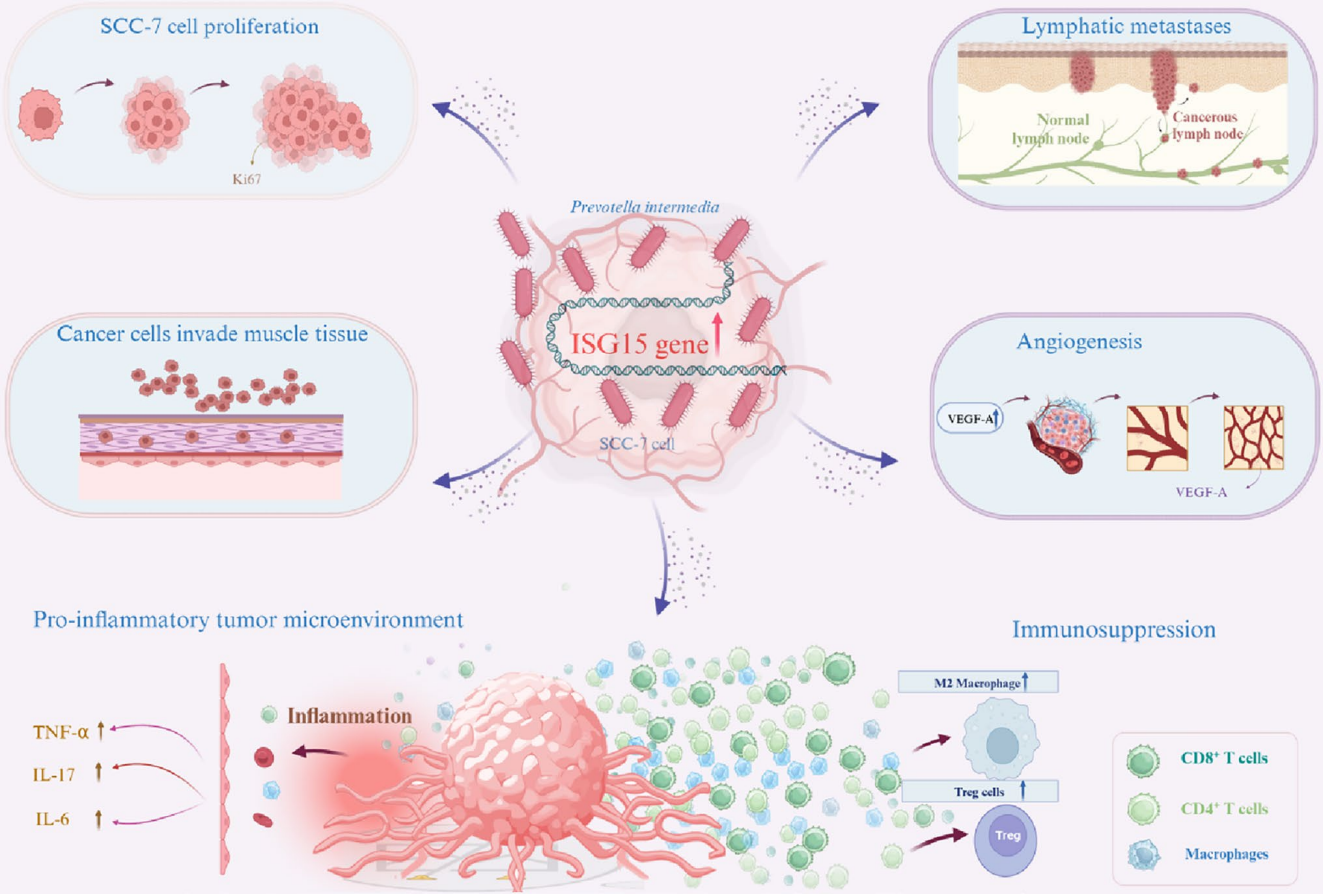
The potential mechanism underlying the effects of *P. intermedia* on OSCC progression

Emerging evidence indicates that oral microbial dysbiosis is common in patients with OSCC (Lindemann et al. 2018). *P. intermedia* is significantly enriched in the mucosal and salivary samples of OSCC patients compared with healthy controls (Heng et al. 2022), and *P. intermedia* infection is associated with an increased risk of oral cancers (Peng et al. 2020). However, evidence of the role of *P. intermedia* in the development of OSCC is still limited. In our study, we used a submucosal xenograft model to assess the effects of *P. intermedia* injection on OSCC progression and development. Our results indicated that *P. intermedia* can promote proliferation, invasion, angiogenesis, and lymph node metastasis in OSCC potentially by upregulating ISG15 (Fig. 7).

ISG15, a ubiquitin-like molecule, which is involved in the occurrence and development of tumors and is closely related to tumor prognosis (Forys et al. 2014). Some investigations have revealed a strong association of high ISG15 expression with the occurrence, development, and metastasis of OSCC (Chairatvit Wongnoppavich and Choonate 2012; Chen et al. 2019; Chi et al. 2009; Laljee et al. 2013; Sumino et al. 2013; Vincent-Chong et al. 2012; Ye et al. 2008; Zhang et al. 2017). Consistently, in our study, *P. intermedia*-induced ISG15 upregulation in xenografts of OSCC was demonstrated by transcriptome sequencing and IHC analysis of tumor tissue. Furthermore, it was found that after the administration of ABX, the expression level of ISG15 was decreased, accompanied by attenuation of malignant phenotypes of OSCC cells, including proliferation, invasion, lymph node metastasis, and angiogenesis. Our results in this study suggest that ISG15 activation caused by *P. intermedia* might play a pivotal role in OSCC progression and malignant behaviors.

Angiogenesis during cancer development is important because the new blood vessel network penetrates and supplies nutrients and oxygen to tumor cells. Several angiogenic factors secreted by tumor cells have been identified, particularly VEGF-A (Cao 2005). VEGF-A plays essential roles in OSCC tumor angiogenesis and metastasis. Consistently, in our study, it was found that the expression level of VEGF-A and microvessel density enhanced after *P. intermedia* infection.

The occurrence of neck lymph node metastasis, the most important prognostic factor in cancer, significantly decreases the survival probability. This explains why OSCC remains one of the tumors with the worst prognosis, with an overall 5-year survival rate of approximately 50% (Pulte and Brenner 2010). Our study indicates that *P. intermedia* infection may play an important role in OSCC progression and metastasis, which may be a potential therapeutic target for OSCC.

IL-6 expression in OSCC has been related to high lymph node metastasis rates and poor tumor differentiation (Chen et al. 2012). IL-6 plays a key role in promoting proliferation and inhibiting apoptosis (Yu Pardoll and Jove 2009). IL-17, as an inflammatory cytokine, supports tumor formation and growth. TNF-α is an inflammatory mediator that has been implicated in carcinogenesis due to its participation in chronic inflammatory diseases (Popa et al. 2007). There is evidence that prolonged TNF-α exposure can increase the proportion of OSCC cells with cancer stem cell phenotypes, which increases their tumor sphere-forming ability (Lee et al. 2012). In a mouse model of ovarian cancer, TNF-α was found to stimulate the secretion of other cytokines, such as IL-17, by CD4^+^ T cells and indirectly promote tumor growth (Charles et al. 2009).

In addition, the relationship between ISG15 expression and immunocyte infiltration in OSCC was analyzed. Chen et al. showed that ISG15 secreted by tumor cells could promote tumor cell migration and immunosuppression in the TME by inducing the polarization of macrophages toward the M2-like phenotype (Chen et al. 2020). Similarly, Tregs in the TME can also impede tumor immune surveillance and suppress antitumor immune responses. Infiltration of a large number of M2 macrophages and Tregs is often associated with poor prognosis (Wang et al. 2023a). In endometrial carcinomas with high expression of ISG15, the abundance of CD8^+^ T cells is significantly reduced and their cytotoxic activity is inhibited, increasing the immune escape ability of tumor cells (Zhao et al. 2022). CD8^+^ T cells are a major subset of T effector cells that can directly target tumor cells and enhance antitumor immune responses. Similarly, CD4^+^ cells may also play an important role in initiating and maintaining anticancer immune responses. In our study, the expression level of ISG15 was positively correlated with the infiltration levels of Tregs and M2 macrophages but negatively correlated with the infiltration levels of CD4^+^ and CD8^+^ T cells.

Currently, there is a plethora of evidence regarding an association between OSCC and chronic inflammation (Rao et al. 2010). The present results suggest that ISG15 may play an important role in the inflammatory and immune microenvironment of OSCC, indicating that ISG15 may be a promising marker of OSCC progression. ISG15, whose expression is aberrantly elevated in OSCC after *P. intermedia* infection, is closely related to numerous malignant behaviors of OSCC cells. Our present study hints at a potential link between *P*. *intermedia* and OSCC progression through ISG15 upregulation. However, our findings only suggest a correlation rather than causation, which is one of the limitations in our present study. Further in vivo and in vitro mechanistic studies such as ISG15 silencing or mouse model of ISG15 deficiency are needed to directly prove causality.

There are still other limitations in our study. First, ABX is not the optimal choice to remove the effects of *P. intermedia* on OSCC progression. It has been shown that orally administered antibiotics can affect both the intratumoral flora and the intestinal flora at the same time (Fu et al. 2022). This complicates the reason why the malignant behavior of tumors is reduced after antibiotic application since it may have an effect on the systemic microbiota of the host in addition to partially eliminating the pro-cancer effects of *P. intermedia*. However, until now, no specific antibiotics for *P. intermedia* have emerged. In future, new and specific chemicals against a particular bacterium may emerge to replace ABX therapy, which may be more helpful to precisely target pathogenic bacteria in the development of OSCC. Besides, OSCC is a malignant tumor with high metastasis and recurrence rates, which may have multiple reasons. Bacterial invasion is only one of the contributing causes. Therefore, potential therapeutic interventions for OSCC are not limited to antibiotics. Some previous studies have also shown that aspirin, in addition to its classical anti-inflammatory function, can also be used as a preventive or therapeutic agent for a variety of cancers, including OSCC (Luo et al. 2021; Zhang et al. 2018). These studies shed a novel light on important insights into non-microbial, pharmacological modulation of akin pathways in OSCC, offering a supplementary viewpoint to our findings on microbial interactions. Therefore, further studies are necessary to deeply investigate the pathways involved in highly malignant behaviors of OSCC and better understand the multifaceted nature of OSCC progression. It’s meaningful to explore the potential molecular targets, which is helpful to find potential therapeutic interventions for OSCC in future.

The feasibility of ISG15 as a molecular biomarker and its prospects for application in the diagnosis and prognosis of OSCC are also worthy of further investigation. ISG15 is likely a prognostic marker and a potential target for OSCC, and its inhibition could provide a therapeutic advantage.

## Conclusion

In summary, our present study uncovered a novel mechanism by which *P. intermedia* promotes OSCC progression and malignant behaviors. The high expression of ISG15 triggered by *P. intermedia* might play a pivotal role in OSCC progression and malignant behaviors. Moreover, finding a new strategy to interfere with *P. intermedia* is an appealing finding and offers new possibilities for the prevention and treatment of OSCC.

## Supplementary Information

Below is the link to the electronic supplementary material.

Supplementary file1 (DOCX 705 KB)

Supplementary file2 (XLS 10 KB)

Supplementary file3 (XLS 5 KB)

## Data Availability

The data that support the findings of this study are available from the corresponding author upon reasonable request.

## Abbreviations

OSCC: Oral squamous cell carcinoma
*P. intermedia*: *Prevotella intermedia*
ABX: Antibiotics cocktail
ELISA: Enzyme-linked immunosorbent assay
IHC: Immunohistochemistry
Tregs: Regulatory T cells
ISG15: Interferon-stimulated gene 15
HNSCC: Head and neck squamous cell carcinoma
ESCC: Esophageal squamous cell carcinoma
CRC: Colorectal cancer
H&E: Hematoxylin–eosin
IF: Immunofluorescence
DMEM: Dulbecco's modified eagle medium
FBS: Fetal bovine serum
BHI: Brain heart infusion broth
PBS: Phosphate–buffered saline
CFU: Colony-forming unit
ROIs: Randomly selected regions of interests
IL: Interleukin
VEGF-A: Vascular endothelial growth factor A
IRS: Immunoreactivity score
FOXP3: Forkhead box protein 3
TNF: Tumor necrosis factor
IFN: Interferon
DEGs: Differential expression genes
GSEA: Gene set enrichment analysis
co-DEGs: Common differentially expressed genes
PPI: Protein–protein interaction network
STRING: Search tool for the retrieval of interacting genes
SDs: Standard deviations of the means
ANOVA: Analysis of variance
TME: Tumor microenvironment
TAMs: Tumor-associated macrophages
ISGs: Interferon-stimulated genes
