# Supplementary material for: Prevotella intermedia boosts OSCC progression through ISG15 upregulation: a new target for intervention
Source: J Cancer Res Clin Oncol. 2024 Apr 21;150(4):206. doi: 10.1007/s00432-024-05730-5 (PMC11033248; doi:10.1007/s00432-024-05730-5)
Supplement: Supplementary file 1 — Supplementary file1 (DOCX 705 KB) [file 432_2024_5730_MOESM1_ESM.docx]

**Supplementary Information**

***Prevotella intermedia* Boosts OSCC Progression Through ISG15 Upregulation: A New Target for Intervention.**

Yao Qin^1^†, Zhiyuan Li^1^†, Ting Liu^1^†, Jingjing Ma^1^, Hong Liu^1^, Yifan Zhou^1^, Suai Wang^1^, Lei Zhang^1^, Qiao Peng^1^, Pei Ye^1^, Ning Duan^1^, Wenmei Wang^1*^, Xiang Wang^1*^.

**Table of Contents**

**Supplementary Figure 1.** Differential expression genes (DEGs) numbers between the control and *P.i* group.

**Supplementary Figure 2.** Clustered heatmaps of DEGs between the *P.i* group and *P.i*+ABX group.

**Supplementary Figure 3.** Gene set enrichment analysis (GSEA) enrichment analysis of the co-expressed genes between the control and *P.i* group .

**Supplementary Figure 4.** Top 10 core gene interaction networks among common differentially expressed genes (co-DEGs) between the control and *P.i* group.

**Supplementary Table 1.** The expression of the upregulated and downregulated DEGs between the control and *P.i* group (*q* ＜ 0.05, FC = 2).

**Supplementary Table 2.** The expression of the upregulated and downregulated DEGs between the *P.i* group and *P.i* ＋ABX group (*q* ＜ 0.05, FC = 2).


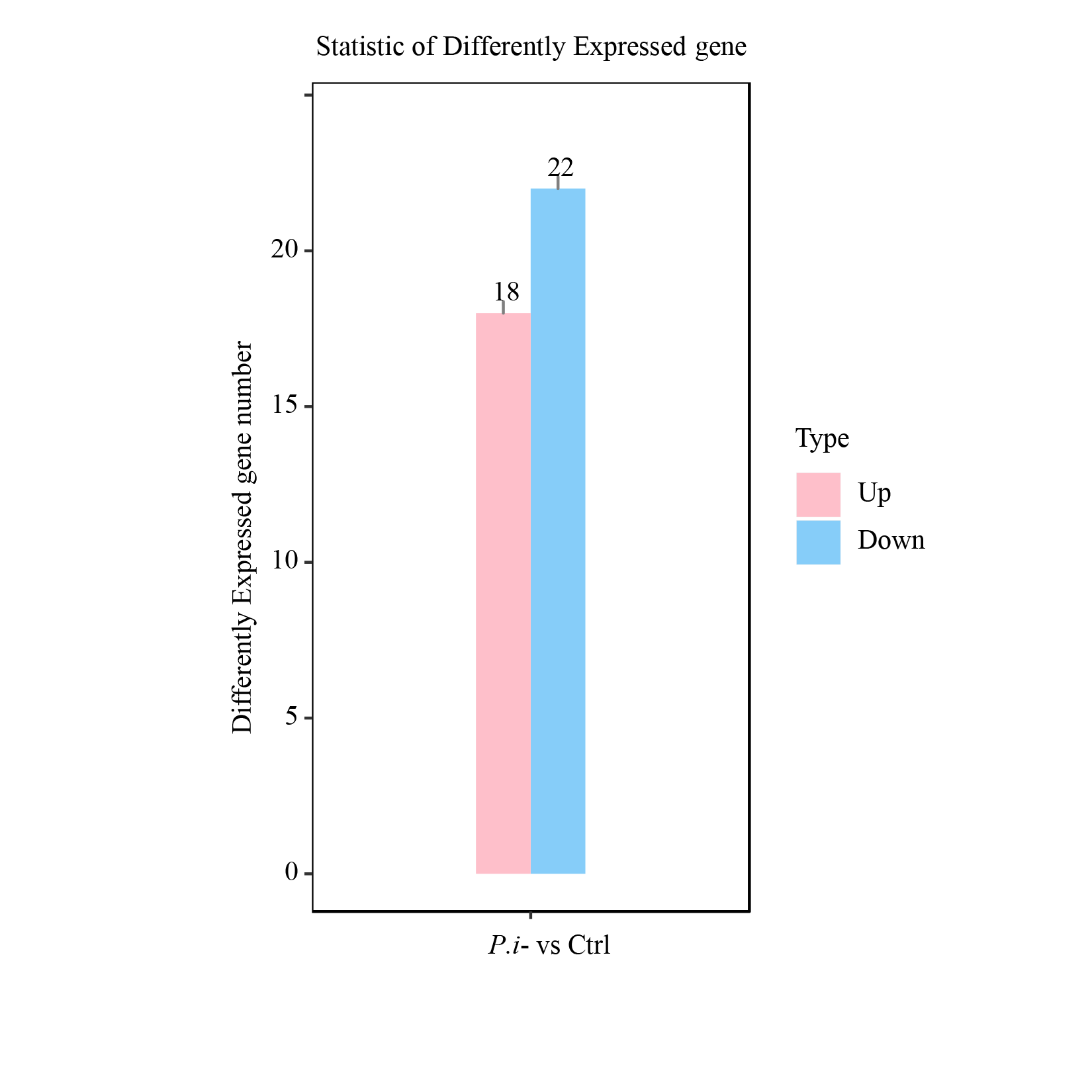


**Supplementary Figure 1.**  **Differential expression genes (DEGs) numbers between the control and *P.i* group.**


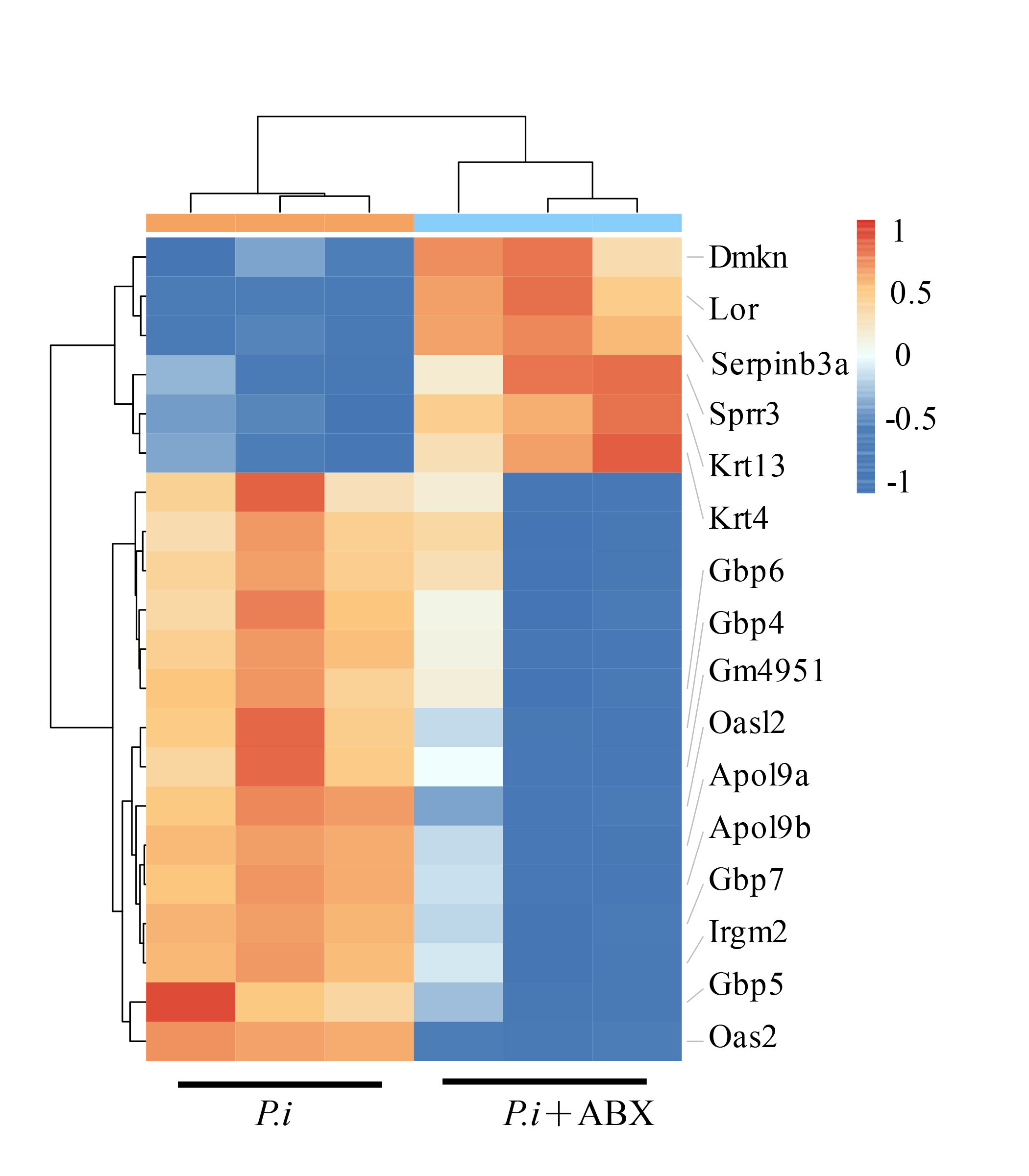


**Supplementary Figure 2. Clustered heatmaps of DEGs between the *P.i* group and *P.i*+ABX group.** Horizontal coordinates represent samples and vertical coordinates represent different genes. Orange represents up-regulated differentially expressed genes and blue represents down-regulated differentially expressed genes.


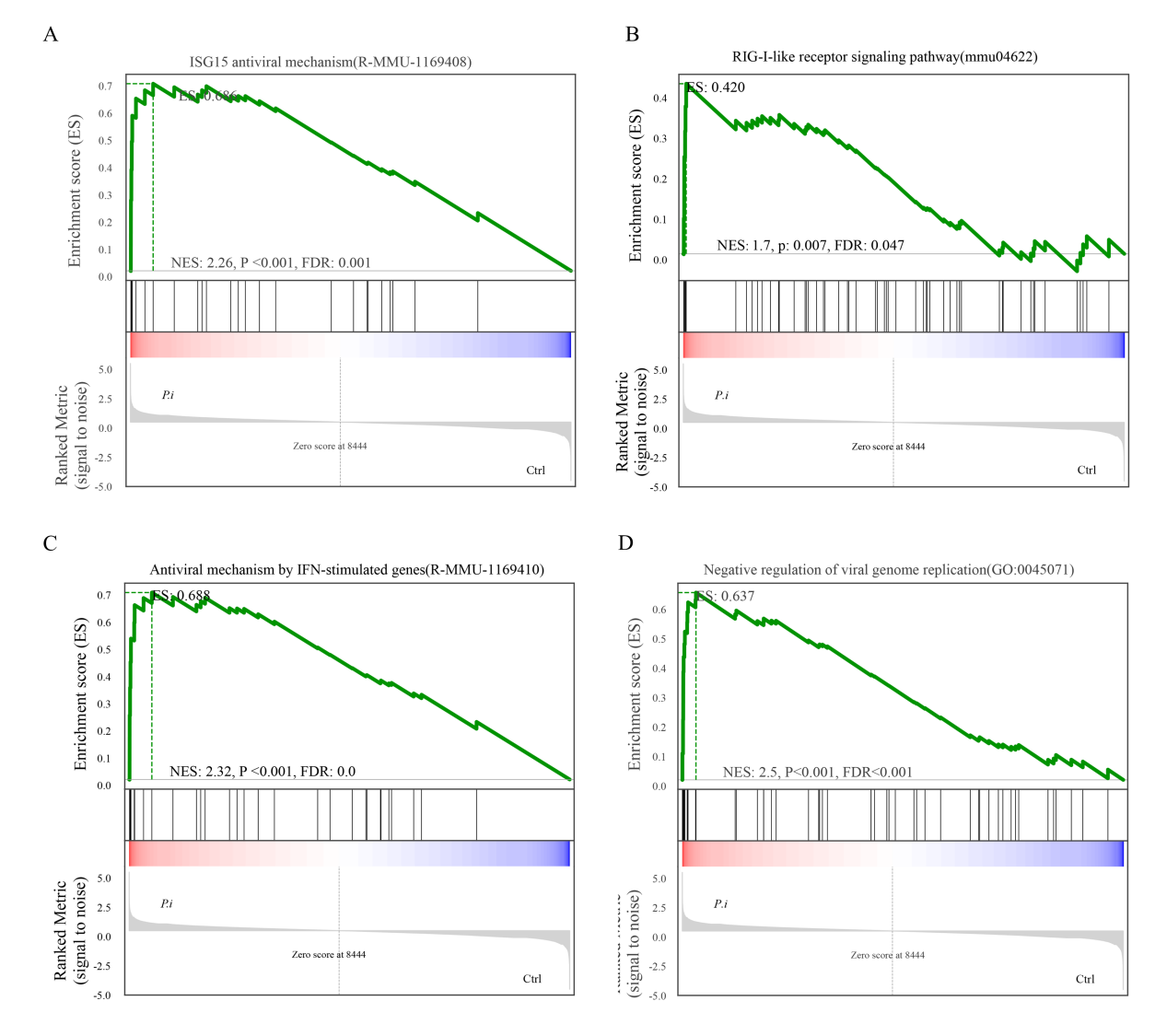


**Supplementary Figure 3. Gene set enrichment analysis (GSEA) enrichment analysis of the co-expressed genes between the control and *P.i* group.** (A) GSEA-based GO analysis of representative gene sets: ISG15 antiviral mechanism. (B) GSEA-based GO analysis of representative gene sets: RIG-I-like receptor signaling pathway. (C) GSEA-based GO analysis of representative gene sets: Antiviral mechanism by IFN-stimulated genes. (D) GSEA-based GO analysis of representative gene sets: Negative regulation of viral genome replication.


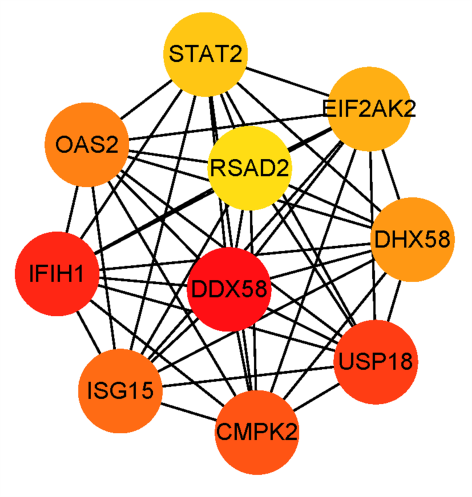


**Supplementary Figure 4. Top 10 core gene interaction networks among common differentially expressed genes** (**co-DEGs) between the control and *P.i* group.** The darker the color, the more powerful the critical degree.
